# A retrospective analysis and case series of glycoprotein IIb/IIIa inhibitor associated diffuse alveolar hemorrhage: two case reports

**DOI:** 10.4076/1757-1626-2-8553

**Published:** 2009-07-30

**Authors:** Hima Mikkilineni, Steven R Bruhl, William R Colyer, Utpal Pandya

**Affiliations:** Division of Cardiovascular Medicine, University of Toledo Medical CenterToledo, OHUSA

## Abstract

**Introduction:**

Glycoprotein IIb/IIIa inhibitors have a key role in the treatment of patients with acute coronary syndromes undergoing percutaneous interventions. Although, an increased risk of bleeding complications is well recognized, its association with diffuse alveolar hemorrhage is much less recognized. Previous authors have suggested that the incidence of glycoprotein IIb/IIIa inhibitor associated diffuse alveolar hemorrhage has been significantly underestimated due to under reporting.

**Case presentations:**

In order to help better determine the incidence of GP IIb/IIIa inhibitor associated DAH, a retrospective review of medical records was conducted over a 1 year period at a single high volume medical hospital. The medical records of all patients diagnosed with diffuse alveolar hemorrhage were evaluated for treatment with a GP IIb/IIIa inhibitor within 48 hours of its diagnosis. Each patient meeting the inclusion and exclusion criteria were included in the case series. This number was compared with the total number of patients receiving a GP IIb/IIIa inhibitor during the same time period and an incidence of the complication was calculated.

292 patients received either abciximab or eptifibatide during the one year review period and two patients were diagnosed with diffuse alveolar hemorrhage confirmed by serial bronchiolar lavage for an incidence of 0.68%. Of the total 292 patients receiving GP IIb/IIIa inhibitors, 172 patients received abciximab with one occurrence of diffuse alveolar hemorrhage for an incidence of 0.58% while 120 patients received eptifibatide with one occurrence for an incidence of 0.83%. Both patients developed significant morbidity as a result of the complication and 1 of the 2 patients died as a complication of the disease.

**Conclusions:**

Our findings support the claim that the incidence of GP IIb/IIIa induced diffuse alveolar hemorrhage is substantially higher than initially suggested by drug manufacturer studies. Although these drugs have proven mortality benefits, its association with diffuse alveolar hemorrhage is likely under-recognized leading to significant under-reporting. The best way to more accurately determine the true incidence of this complication and decrease its morbidity and mortality is to increase awareness as well as include diffuse alveolar hemorrhage as a serious complication in product labeling.

## Introduction

Glycoprotein IIb/IIIa (GP IIb/IIIa) inhibitors are potent antiplatelet agents with proven benefits in the setting of high-risk percutaneous coronary interventions (PCI). Abciximab (ReoPro) is a fab fragment immunoglobulin that binds to platelet glycoprotein IIb/IIIa receptors preventing platelet aggregation and thrombus formation [1]. Likewise, eptifibatide (Integrilin), is a cyclic heptapeptide that selectively binds to the glycoprotein IIb/IIIa receptor with similar effects on platelet binding. Both have been proven to have short and long-term benefits in clinical studies [1,4]. In a meta-analysis of the effectiveness and safety of GP IIb/IIIa receptor blockers used in PCI, Brown et al showed that treatment with eptifibatide was not associated with a significant increase in risk of major bleeding. However, a more recent case series suggested that this complication is likely significantly underestimated partially due to the fact that no GP IIb/IIIa receptor blockers list pulmonary hemorrhage as associated complications of their use [2].

This retrospective analysis was initiated in order to better determine the incidence of GP IIb/IIIa associated DAH. We also sought to identify risk factors which might increase a patients risk of developing DAH.

## Case presentations

### Methods

A retrospective review of medical records was conducted at the University of Toledo Medical Center in Toledo, OH, a level 1 trauma center with 3 high volume cardiac catheterization suites. All patients that met each of the inclusion and exclusion criteria between July 2006 and 2007 were included in the study.

The numbers of patients receiving at least one dose of GP IIb/IIIa inhibitor were identified through a computer search of the hospital pharmacy’s dispensing records during the designated review period.

Patients diagnosed with diffuse alveolar hemorrhage were identified through a computer search of the hospitals database between July 2006 and June 2007. Each chart was then reviewed using the following inclusion and exclusion criteria.

### Inclusion Criteria

1. New diagnosis of DAH confirmed by serial bronchoalveolar lavages.
2. Treatment with any Glycoprotein IIb/IIIa inhibitor within 7 days of the diagnosis of DAH.

### Exclusion Criteria

1. Treatment with any drugs known to be associated with DAH within the previous 10 days other than a GP IIb/IIIa inhibitor.
2. Diagnosis of Wegener’s granulomatosis, Goodpasture’s syndrome, idiopathic pulmonary hemosiderosis, collagen vascular disease or microscopic polyangiitis.

Follow-up: All patients with DAH were evaluated by both a Cardiologist and a Pulmonary Critical Care Specialist on a daily basis throughout their hospitalization.

## Results

292 patients received either abciximab or eptifibatide during the one year review period and revealed two patients diagnosed with diffuse alveolar hemorrhage confirmed by serial bronchiolar lavage for an incidence of 0.68%. Of the total 292 patients receiving GP IIb/IIIa inhibitors, 172 patients received abciximab with one occurrence of diffuse alveolar hemorrhage for an incidence of 0.58% while 120 patients received eptifibatide with one occurrence for an incidence of 0.83%. Both patients developed significant morbidity as a result of the complication and 1 of the 2 patients died as a complication of the disease.

### Case report 1

**Figure 1.**
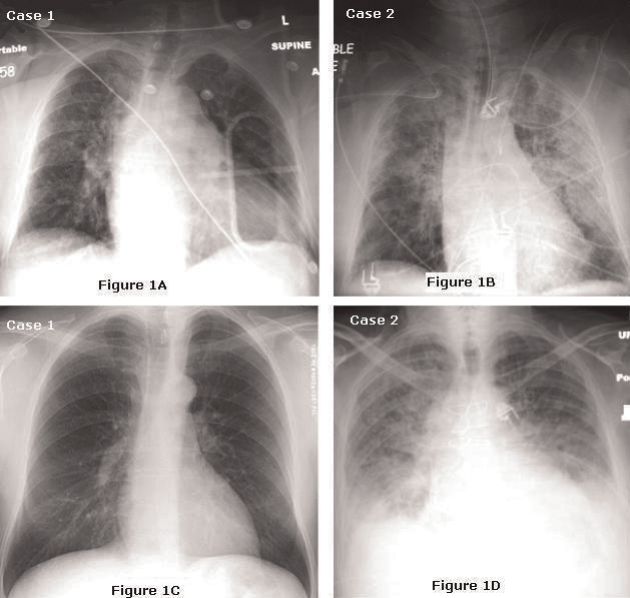
CXR findings in GP IIb/IIIa induced DAH. **(A & B)** Anteroposterior view of chest x-ray at presentation. **(C)** Anteroposterior view of chest x-ray seven days later. **(D)** Anteroposterior view of chest x-ray day later.

A 72 year old white female was transferred to the emergency room by life-flight complaining of left sided chest pain. Her past medical history was significant for hypertension, hypothyroidism and hyperlipidaemia and the patient had no prior history of smoking, cardiac disease, or lung disease. The patients blood pressure at presentation was 153/83, pulse 68 and respirations 20 per minute. The patient denied any significant shortness of breath, but her presenting chest X-ray (CXR) showed mild bilateral alveolar infiltrates consistent with mild congestive heart failure (Figure 1) and electrocardiography (ECG) showing anterior ST elevations consistent with an acute myocardial infarction. Laboratory evaluation included a complete blood count, electrolytes, and coagulation profile which were all within normal limits. The patient was treated with aspirin, intravenous nitro-glycerine, morphine and heparin by weight based protocol. Beta blockers were held secondary to a mild hypotension into the low 100’s after administration of morphine and IV nitro-glycerine. Within 45 minutes of arrival, the patient was taken emergently to the cardiac catheterization laboratory for primary percutaneous revascularization. Her angiogram revealed a proximal 99% stenosis of the left anterior descending artery which was treated with balloon angioplasty and stent placement. This resulted in a complete reduction of the stenosis with corresponding TIMI-3 flow in the affected artery. However, before intervention, the patient was treated with a 0.25 mg/kg bolus of abciximab, followed by continuous intravenous infusion at 0.125 mcg/kg/min for 12 hours. Due to the patients’ mild to moderate hypotension, she also underwent right heart catheterization which showed a right atrial pressure of 10, right ventricle pressure of 35/8-10, pulmonary artery pressure of 35/25, pulmonary capillary wedge pressure of 25, cardiac output 4.18 litres/min and cardiac index of 2.21 litres/min/m^2^. Based on these findings the decision to place a temporary intra-aortic balloon pump was made.

Approximately 30 minutes following the procedure, the patient developed acute shortness of breath with pending respiratory failure requiring emergent oral intubation. Prior to intubation, the anaesthesiologist reported observing bright red blood within the posterior oropharynx, as well as some blood within the endotracheal tube after its placement. CXR immediately following intubation showed moderate to severe bilateral alveolar infiltrates which were a marked change from her admission CXR (Figure 1A & 1C). These findings were attributed to acute cardiogenic pulmonary edema and was therefore started on intravenous furosemide and continued on intra-aortic balloon pump support. However, over the first 12 hours of the patient’s admission, she was noted to have a 3.2 gram drop in haemoglobin of an unknown etiology. This lead to the transfusion of two units of packed red blood cells. However, although the patient’s hemodynamic status improved allowing the discontinuation of the balloon pump and heparin, over the next 72 hours the patient developed diffuse alveolar infiltrates with worsening hypoxia. Given this clinical picture, the diagnosis of DAH was suspected and the patient underwent bronchoscopy. This showed dark blood throughout the bronchial tree with increasing hemorrhagic return on serial bronchiolar lavage confirming the diagnosis. In spite of intensive supportive care over two additional weeks, the patient condition continued to deteriorate and she died on hospital day 21.

### Case report 2

A 82-year-old man with the diagnosis of unstable angina was transferred for possible PCI. His past medical history included hypertension, hyperlipidemia, chronic renal insufficiency (baseline creatinine 2 mg/dl), and previous myocardial infarction with five vessel CABG for coronary artery disease 10 years ago. He did have a remote smoking history of four years but quit 65 years ago and had no known history of lung disease.

Upon arrival to our facility, he was pain free and his blood pressure was 120/75 mmHg with heart rate of 74 beats per minute. ECG showed normal sinus rhythm, left axis deviation, and ST segment depression in the inferior leads. The patients’ prothrombin time was 14 seconds with a corresponding INR of 1.2 and a normal platelet count. CXR did not reveal any infiltrates or other acute processes (Figure 1B).

The patient was treated with full dose aspirin, started on heparin by standard weight based protocol, and taken to the cardiac catheterization laboratory for coronary angiography. The coronary angiogram showed severe native three vessel disease, widely patent RIMA and LIMA, and 80% focal stenosis in the mid portion of the patients saphenous vein graft to the obtuse marginal branch of the left circumflex artery. These findings correlated with the patients ECG findings and therefore he underwent percutaneous coronary intervention of his saphenous vein graft. A bolus of 180 mcg/kg of intravenous eptifibatide was given followed by a continuous intravenous infusion at 1 mcg/kg/min for 18 hours. Balloon angioplasty and stenting was performed using standard technique and reduced the stenosis to 0% restoring TIMI-3 coronary flow to the vessel. Following the procedure, the patient was transferred to the coronary care unit in stable condition.

Four hours following the procedure the patient developed severe dyspnea and hypoxemia. Electrocardiography revealed sinus tachycardia and arterial blood gas measurements on 100% non re-breather mask showed a pH of 7.44, Po_2_ of 50 mmHg a Pco_2_ of 32 mmHg, and oxygen saturation of 85%. Chest examination revealed diffuse rales over both the lung fields, and bed side CXR revealed bilateral diffuse alveolar infiltrates. (Figure 1D).

Based on the clinical picture, the patient was diagnosed with respiratory insufficiency secondary to acute cardiogenic pulmonary edema related to his recent myocardial infarction and was treated with intravenous diuretics for 48 hours. During this time, the patients clinically picture did not improve leading to a right heart catheterization revealing a right atrial pressure of 6 mmHg, a pulmonary capillary wedge pressure of 9 mmHg, cardiac output of 5.45 litres/min, and a cardiac index of 2.74 litres/min/sqm. These findings were suggestive of non-cardiogenic pulmonary edema and resulted in an alternative workup for the patients’ unexplained respiratory insufficiency and pulmonary infiltrates. The possibility of pulmonary embolus was first investigated, but negative lower extremity Doppler’s and a low probability ventilation perfusion scan made this diagnosis unlikely.

**Figure 2.**
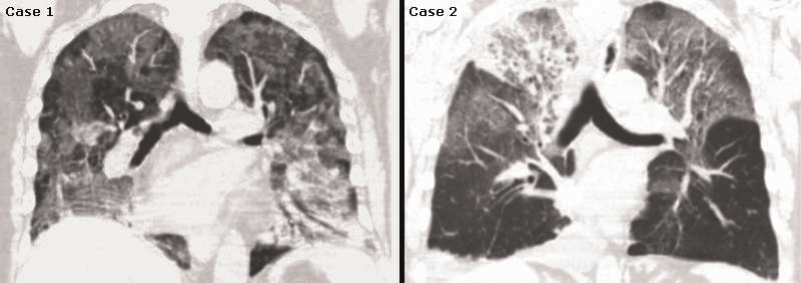
CT scan findings in DAH, Sagittal high resolution chest CT without contrast showing bilateral ground glass opacities with interstitial septal thickening suggestive of acute interstitial process.

High resolution chest computed tomography was performed without contrast and showed bilateral ground glass opacities with interstitial infiltrates involving the upper and lower lobes consistent with pulmonary edema (Figure 2).

When the patients’ haemoglobin subsequently fell two gram over 24 hours, the diagnosis of DAH was suspected. The patient underwent bronchoscopy which revealed diffused blood throughout the bronchial tree with increased hemorrhagic return on serial bronchiolar lavages confirming the diagnosis of DAH. Subsequently, the patient was treated with methylprednisolone 125 mg IV × 3 days followed by a 9 day taper. The patients oxygenation steadily improved over the next 7 days and the patient was eventually discharged from the hospital on day 17 days with no further complications.

## Discussion

Glycoprotein IIb/IIIa inhibitors are antiplatelet agents currently recommended in the treatment of patients with unstable angina and non ST-elevated myocardial infarctions [3]. There efficacy is believed to be secondary to reduced platelet aggregation as well as decreased thrombin generation and fibrin formation associated with PCI. However, despite these clear cardiovascular benefits, there is a coexisting increase in the risk of bleeding complications. Increased bleeding complications are most commonly seen at vascular access sites, however, other reported bleeding sites include intra-cranial, retroperitoneal, urological and gastrointestinal sources [4].

Sitges and Villa were the first to report a case of pulmonary hemorrhage after abciximab use in 1997, and more recently *Orford* reported the first case of eptfibatide associated alveolar hemorrhage in 2004 [5]. Since then, there has been a scattered, but steadily growing number of case reports detailing accounts of alveolar hemorrhage associated with the use of all three GP IIb/IIIa inhibitors.

Early diagnosis of pulmonary hemorrhage can be extremely challenging. This is because symptoms such as hemoptysis, hypoxemia, and new chest radiological infiltrates are often erroneously attributed to congestive heart failure, acute respiratory distress syndrome, or pulmonary embolism. The fact that hemoptysis can be absent in as many as one-third of all cases of DAH further complicates the diagnosis [6]. Misdiagnosis usually results in inappropriate evaluation and treatment of suspected heart failure, unnecessary use of antibiotics, or even extended exposure to anticoagulation. Since the diagnosis of DAH from any cause represents a medical emergency, prompt diagnosis and removal of the inciting agent is of utmost importance.

Recently Khanlou et al reported seven cases of pulmonary hemorrhage associated with the use of abciximab and suggested that the presence of underlying lung conditions, such as chronic obstructive pulmonary disease, pulmonary hypertension, high pulmonary-capillary wedge pressure, and elevated left ventricular end diastolic pressure (LVEDP) may be associated with an increased risk of pulmonary hemorrhage [6]. In our case series, elevated LVEDP and high pulmonary-capillary wedge pressures were present and old age, chronic renal insufficiency, and standard doses of heparin used in combination with GP IIb/IIIa inhibitors may have increased the patient’s risk of developing pulmonary hemorrhage. These cases are part of a growing body of evidence that suggest the incidence of GP IIb/IIIa induced pulmonary hemorrhage may be greater than previously reported due to under-recognition or misdiagnosis. Those researchers also suggested that failure of GP IIb/IIIa inhibitor manufacturers to include this complication in its product labeling likely contributes to under diagnosis of this complication.

A recent large scale retrospective analysis of patients receiving abciximab or eptifibatide reported that alveolar hemorrhage was diagnosed in 11 of 5458 (0.2%) treated patients compared to only one episode of alveolar hemorrhage in the 4136 patients (0.025%) not receiving GP IIb/IIIa inhibitors. Compared to control group, patients treated with GP IIb/IIIa inhibitors had a statistically significant increase in the risk of developing alveolar hemorrhage.

Although there are no established guidelines for the treatment of GP IIb/IIIa receptor inhibitor induced alveolar hemorrhage, removal of the offending agent as well as discontinuation of all forms of anticoagulation as soon as possible remains the mainstay of treatment. Although the use of penacillamine induced alveolar hemorrhage has been shown to benefit from treatment with corticosteroids, most other drug induced causes of alveolar hemorrhage have not been shown to benefit from its use. Therefore, the decision to use steroids in GP IIb/IIIa inhibitor induced alveolar hemorrhage is controversial and varies by clinician.

Although the patient in our second case received steroids and subsequently improved, while the patient not treated with steroids failed to recover; one should not prematurely conclude that a correlation between these two things exists. Although both developed DAH in association with GP IIb/IIIa inhibitors, the differences between these two cases were substantial. More likely, the adverse outcome in the first case may simply be related to the fact that the diagnosis was delayed over 72 hours longer than that of the second patient (Table 1).

**Table 1.** Comparison of Patients diagnosed with DAH

Variables	Case 1	Case 2
Age	72	82
Gender	F	M
Glycoprotein IIb/IIIa	abciximab	eptifibatide
Hours till diagnosis	122	50
Treatment received	supportive	supportive + steroids
Outcome	died day 21	discharged day 17

## Conclusions

A high index of clinical suspicion for DAH is essential whenever GP IIb/IIIa inhibitors are used. Respiratory distress, worsening alveolar infiltrates, in addition to a sudden fall in haemoglobin with or without hemoptysis, should alert the physician to the possibility of pulmonary hemorrhage. Diagnostic bronchoscopy should be considered early when the diagnosis is suspected because adverse outcomes seem to correlate with delayed diagnosis and treatment. Current treatment of DAH from any cause includes discontinuation of offending agents, stopping all anticoagulation as soon as possible, and careful consideration of the potential risks and benefits of using intravenous corticosteroids [3].

The diagnosis of DAH is a serious condition that is associated with significant morbidity and mortality. Since early diagnosis appears key to decreasing adverse outcomes, we agree with the conclusions of Iskandar in stating that diffuse alveolar hemorrhage is likely an under-diagnosed complication of GP IIb/IIIa inhibitors and that this complication should be added to the product labelling of GP IIb/IIIa inhibitors as a potentially life threatening adverse reactions.

## Abbreviations

CABG: Coronary artery bypass grafting
CXR: chest X ray
DAH: Diffuse alveolar hemorrhage
ECG: Electrocardiography
GP: Glycoprotein
IV: Intravenous
LIMA: Left internal mammary artery
PCI: Percutaneous coronary intervention
RIMA: Right internal mammary artery
TIMI: Thrombolysis in myocardial infarction
